# Meteorological Table

**Published:** 1811-12

**Authors:** 


### METEOROLOGICAL TABLE.

*From October 26, to November 26.*

| | D | Therm. | | | Barom | | Hygrom. dry——damp | | | Winds. | Atmos. Variation. |
|---|---|---|---|---|---|---|---|---|---|---|---|
| | 27 | 48 | 53 | 50 | $29^{9}$ | — | 20 | — | 23 | SE . . | F . . — . . . . R . . F . . |
| | 28 | 49 | 53 | 48 | $28^{9}$ | —$^{8}$ | 21 | — | 24 | E . . | F . . . — . . R . . . . |
| | 29 | 46 | 52 | 50 | $28^{9}$ | $29^{2}$ | 22 | 20 | 22 | NW . . . | R . . F. R. F. . . . R . . . |
| | 30 | 50 | 55 | 53 | $29^{1}$ | — | 23 | 30 | — | S . . | R . . . F. R . . . . |
| ○ | 31 | 48 | — | 51 | $29^{5}$ | —$^{8}$ | 26 | — | 30 | SW . . | F . . — . . . R . — . . . |
| | 1 | 58 | 60 | 61 | $29^{7}$ | — | 32 | 38 | 35 | S . . . | C . . . . R . . . — . . . . |
| | 2 | 62 | 65 | 61 | $29^{7}$ | —$^{5}$ | 42 | — | 44 | SW . . . | C . . . . R . . — . . . |
| | 3 | 56 | 57 | 54 | $29^{6}$ | —$^{7}$ | 36 | 30 | 30 | SW . . | F . . . C . . R . . . in N. |
| | 4 | 53 | 56 | 49 | $29^{7}$ | 30 | 30 | 28 | 25 | SW . . . | F . . . R. F. . . . |
| | 5 | 53 | — | 50 | $29^{8}$ | —$^{9}$ | 33 | — | 32 | W . . . | R . . — . . . F . . . |
| | 6 | 49 | 53 | 51 | $29^{9}$ | —$^{6}$ | 30 | — | 36 | W . . | R , C . . . R . . . . — . . . |
| ◑ | 7 | 50 | — | 47 | $29^{5}$ | —$^{6}$ | 35 | 23 | 30 | W . . | F . . . — . . R . . . in N. |
| | 8 | 45 | 49 | 51 | $29^{5}$ | —$^{4}$ | 26 | 30 | 36 | E . . | C . . . R . . — . . . C . . . . |
| | 9 | 49 | 53 | 52 | $29^{6}$ | —$^{7}$ | 36 | 31 | 35 | SE . . | C . . . . F . . C . . . . |
| | 10 | 53 | 52 | 49 | $29^{4}$ | —$^{3}$ | 38 | 26 | 33 | SW . . | R . . . . F . . . R . . . |
| | 11 | 45 | 51 | 47 | $29^{3}$ | —$^{5}$ | 30 | — | 32 | W . . . | F . . . C . . . F . . . |
| | 12 | 41 | 51 | 45 | $29^{9}$ | —$^{8}$ | 30 | — | 33 | NW . | F . . . C . . . F . . R . . . . |
| | 13 | 51 | 50 | 43 | $29^{5}$ | —$^{7}$ | 38 | 28 | 30 | W . . | F . . . R . . F . . . . |
| | 14 | 43 | 53 | 45 | $29^{7}$ | — | 30 | 33 | 26 | W . . | F . . R . . F . . . . |
| ● | 15 | 43 | 44 | 39 | $29^{6}$ | —$^{5}$ | 28 | 17 | 25 | W . . . | F . . . . — . . . . R . . in N |
| | 16 | 42 | 47 | 44 | $29^{5}$ | —$^{8}$ | 26 | — | 24 | NW . . . | F . . . — . . — . . . . |
| | 17 | 43 | 46 | — | 30 | —$^{2}$ | 24 | — | 25 | N . . | F . . . C . . R . . C . . . . |
| | 18 | 48 | 50 | 48 | $30^{2}$ | —$^{3}$ | 36 | 30 | 25 | W . | Fog . . . R . . F . . . |
| | 19 | 48 | 50 | 44 | $30^{2}$ | —$^{3}$ | 30 | 24 | 19 | W . . | C . . . F . . . — . . . C . . . |
| | 20 | 41 | 54 | 41 | $30^{3}$ | — | 26 | 20 | 21 | N . . | F . . . — . . . . C . . . |
| | 21 | 37 | 42 | 40 | $30^{3}$ | — | 21 | 20 | 21 | NW . | Fog . . . F . . C . . |
| | 22 | 40 | 43 | 40 | $30^{2}$ | — | 22 | 19 | 22 | NE . . | Fog . . . F . . — . . . . |
| ◐ | 23 | 33 | 40 | 38 | $30^{2}$ | — | 21 | — | — | N . | Fog . . . — . . F . . |
| | 24 | 38 | 44 | 40 | $30^{2}$ | — | 21 | 25 | 27 | NW . . | F . . . — . . . . C . . |
| | 25 | 40 | 48 | 46 | $30^{3}$ | — | 23 | 26 | 26 | NW . | F . . . C . . . R . F . . . |
| | 26 | 44 | 46 | 45 | $30^{3}$ | —$^{4}$ | 24 | 20 | 19 | NW . | C . . . F . C . . . |

Quantity of rain from October the 26th to November the 26th, 3 inches $\frac{33}{100}$. On twenty days of this interval there was rain, and on four days very considerable fog. On the 23d and morning of the 24th, stagnant waters in the neighbourhood of London were covered with ice.

*Princes Street, Cavendish Square.*

*Printed by E. Hemsted, Great New Street, Fetter Lane.*

